# Supplementary material for: Risk factors and visual outcome of Non-Arteritic Ischemic Optic Neuropathy (NAION): Experience of a tertiary center in Kuwait
Source: PLoS One. 2021 Feb 18;16(2):e0247126. doi: 10.1371/journal.pone.0247126 (PMC7891726; doi:10.1371/journal.pone.0247126)
Supplement: Attachment — Submitted filename: Behbehani PONE final review 11521.docx [file pone.0247126.s001.docx]

Behbehani PONE-D-20-30757R1: Risk Factors and Visual Outcome of NAION in Kuwait

The paper has bad spelling, grammar, to the point that it is painful to read. I STRONGLY recommend that the manuscript should be reviewed by someone whose primary language is English before this is resubmitted! There are some important data here but it is overshadowed by non-scientific issues! There are also problems with both understanding of facts and logic! Please see my other comments below.

Abstract:

Results paragraph-Young for ‘Yong’

Conclusion: ‘**A** high prevalence’ rather than ‘high prevalence’

Introduction:

1. NAION is not the most common optic neuropathy. **Glaucoma is the most common optic neuropathy** (it affects 60,000,000 people in the world, vs ~10,000,000 max for NAION, assuming an incidence of 10.2/100,000 for NAION). NAION is the second most common optic neuropathy and the most common cause for sudden optic nerve-related vision loss.
2. Put the reference numbers (1); ((2,3) before the period at the end of the sentence.
3. ….optic nerve head resulting IN ischemia….
4. **Associated** with a small cup-to-disc ratio (it does not explain why predisposed individuals frequently have a small cup-to-disc ratio).
5. Inappropriate work-**up** and management.
6. …NAION **who were** referred to a tertiary care center…
7. younger NAION **patients (young)**, not younger NAION (Young)….
8. older NAION **patients** (not older NAION)

Methods:

Statistical analysis: …to determine if there **WAS** any independent effect….

Results: The paragraph describing visual outcome in young vs old NAION is confusing and should be revised (and this should be written: young patients (or individuals) vs old patients (or individuals) with NAION). The last sentence in this paragraph is describing the **MEAN** final logmar visual acuity for Young individuals with NAION and **MEAN** final logmar visual acuity for old individuals with NAION.

Visual Field improved in 40.6%	Visual Field improved in 28.3%

Stable in 46.9%	Stable in 60.9%

Worsened in 12.5%	Worsened in 10.9%

There was a trend for higher prevalence (remove the ‘However’)

In young **patients** (or individuals) with NAION (not ‘in young NAION’)

In older **patients** with NAION (not ‘older NAION’)

**Discussion:**

The authors need to clarify their discussion of the effect of improved **mean** acuity in the young individuals with NAION (paragraph 2). The reason for this is the difference between the **number** of younger individuals who improve their acuity, vs the MEAN final acuity. There is already a trend for better acuity in young individuals with NAION vs older individuals with NAION (Baseline mean logMAR visual acuity; p value=0.17)

**Young NAION patients**:	**Old NAION patients**:

**Visual acuity improved in 21.9% (n=7),**	**Visual acuity improved in 39% (n=18)**

Stable in 56.3% (n=18)	Stable in 33.6% (n=15)

Worsened in 21.9% (n=7)	Worsened in 28.3% (n=13)

Visual acuity improved in more older NAION patients than in younger patients, **but the number of older patients whose visual acuity improves (n=18/46 total) is still just a trend (n=7/32 total) (Z-score calculation for two population proportions https://www.socscistatistics.com/tests/ztest/default2.aspx)**

**Final MEAN LogMar visual acuity:**

0.55±0.57	0.9±0.73

That the ULTIMATE MEAN visual acuity in the younger age group is better does not mean that the outcome for any younger individual is more favorable, since fewer younger individuals have improvements in their visual acuity. The authors are clearly trying to puzzle this out with their second paragraph, with their inclusion of Sun, Hayreh and IONDT data.

What should one tell their patients? That they are likely to have a worse chance of visual improvement if they are young (21.9% in young vs 39% for old), or that they are likely to have a better mean final visual acuity, or that since they are young, they are more likely to have a better starting acuity?

Importantly, the authors have made a crucial error in their third paragraph. A cup to disk ratio <0.3 cannot be seen in 61.5% of patients overall when you have 31% of young individual with a c:d <0.3 and 43% of older individuals with a c:d<0.3. The total number of individuals was 78; the total number of individuals with C:D< 0.3 is 30; the percentage of total with C:D<0.3 is 38.4% (30/78).
