# Supplementary material for: Risk factors and visual outcome of Non-Arteritic Ischemic Optic Neuropathy (NAION): Experience of a tertiary center in Kuwait
Source: PLoS One. 2021 Feb 18;16(2):e0247126. doi: 10.1371/journal.pone.0247126 (PMC7891726; doi:10.1371/journal.pone.0247126)
Supplement: Attachment — Submitted filename: Answers to reviewer Comments-R2.docx [file pone.0247126.s002.docx]

Answers to reviewer Comments

Reviewer 1:

1) The paper has bad spelling, grammar, to the point that it is painful to read. I STRONGLY recommend that the manuscript should be reviewed by someone whose primary language is English before this is resubmitted!

Answer: The paper was revised extensively again for syntax, grammar and spelling errors and the errors pointed out by the reviewer and other errors found in the text were corrected.

2) **Glaucoma is the most common optic neuropathy** (it affects 60,000,000 people in the world, vs ~10,000,000 max for NAION, assuming an incidence of 10.2/100,000 for NAION). NAION is the second most common optic neuropathy and the most common cause for sudden optic nerve-related vision loss.

Answer: Agree. We have added the sentence suggested by the reviewer.

3) Put the reference numbers (1); ((2,3) before the period at the end of the sentence.

Answer: This was changed throughout the text.

4) Results: The paragraph describing visual outcome in young vs old NAION is confusing and should be revised (and this should be written: young patients (or individuals) vs old patients (or individuals) with NAION). The last sentence in this paragraph is describing the **MEAN** final logmar visual acuity for Young individuals with NAION and **MEAN** final logmar visual acuity for old individuals with NAION.

Answer: This was changed and corrected as suggested by the reviewer.

5) The authors need to clarify their discussion of the effect of improved **mean** acuity in the young individuals with NAION (paragraph 2). The reason for this is the difference between the **number** of younger individuals who improve their acuity, vs the MEAN final acuity. There is already a trend for better acuity in young individuals with NAION vs older individuals with NAION (Baseline mean logMAR visual acuity; p value=0.17). **Young NAION patients**:	**Old NAION patients**:

**Visual acuity improved in 21.9% (n=7),**	**Visual acuity improved in 39% (n=18)**

Stable in 56.3% (n=18)	Stable in 33.6% (n=15)

Worsened in 21.9% (n=7)	Worsened in 28.3% (n=13)

That the ULTIMATE MEAN visual acuity in the younger age group is better does not mean that the outcome for any younger individual is more favorable, since fewer younger individuals have improvements in their visual acuity. The authors are clearly trying to puzzle this out with their second paragraph, with their inclusion of Sun, Hayreh and IONDT data.

Answer: We thank the reviewer for this comment. I think we were responsible for this confusion since the we have the percentages above was part of overview descriptive statistics and ANY change in visual acuity even single Snellen line or 0.1 logMAR unit was calculated as improvement in visual acuity. This has to led to over-estimation of improvement or worsening in visual acuity and was reflected by having seemingly more improvement in visual acuity in older NAION patients. However, when we re-calculated this based on the criteria of for visual acuity improvement presented in the methods section (improvement of visual acuity of at least 0.3 logMAR unit), we have found that slightly more of young NAION patients actually had improved visual acuity and the majority of patients in both young and older NAION patients was stable. The revised calculation is the following:

| LogMAR acuity change | Young NAION patients | Older NAION patients |
| --- | --- | --- |
| Better | 11 (34%) | 12 (26%) |
| Stable | 17 (53.2%) | 25 (54.3%) |
| Worse | 4 (12.5%) | 8 (19.3%) |

Similarly, when have calculated a change of MD visual field as at least 3 decibel change to qualify for either improvement or worsening in MD, we found the following:

| MD visual field change | Young NAION patients | Older NAION patients |
| --- | --- | --- |
| Better | 13 (40.6%) | 12 (26.3%) |
| Stable | 15 (46.9%) | 28 (60.0%) |
| Worse | 4 (12.5%) | 6 (13%) |

Therefore, despite having a trend for younger NAION patients for a better presenting visual acuity compared to older NAION patients, more of the young NAION patients had an improvement of logMAR visual acuity than older NAION patients from baseline to follow up (34% vs 26%) but this was not statistically significant. Similarly, more of the young NAION patients had improvement of the visual field MD than older NAION patients from baseline to follow up (40.6% vs 26.3%) but this was also not statistically significant. Only when we used a final visual acuity of 20/40 or better as an outcome, young NAION patients reached this outcome significantly more than older NAION patients. Finally, there was a trend towards young NAION patients having a better visual field MD at follow up than older NAION patients (-11.52 vs 14.85, p=0.1).

Therefore, in trying to answer the question posed by the reviewer as to whether young NAION patients have a better final visual acuity because there was a trend towards in them having a better presenting visual acuity, this certainly may one valid hypothesis. However, at least by numbers, young NAION patients also they also were more likely to improve in visual acuity (34% vs 26%) and visual field MD (40.6% vs 26.3%) from baseline to follow up. Therefore, it is quite possible that they there is an element both factors (better presenting visual acuity and higher likelihood of improvement over the course of NAION in younger patients) but our study was not powered enough for this to reach statistical significance. We have added this clarification in the discussion and we have acknowledged this as a limitation of our study. We have updated table 2 to include these comparisons. Finally, we have removed the sentences in the discussion citing the paper Hayreh and the IODNT which we felt they were strictly non-relevant to our findings.

6) Importantly, the authors have made a crucial error in their third paragraph. A cup to disk ratio <0.3 cannot be seen in 61.5% of patients overall when you have 31% of young individual with a c:d <0.3 and 43% of older individuals with a c:d<0.3. The total number of individuals was 78; the total number of individuals with C:D< 0.3 is 30; the percentage of total with C:D<0.3 is 38.4% (30/78).

Answer: We thank the reviewer for this comment. This has unfortunately stemmed from an inadvertent error in Table 2 which we have corrected since the total number of patients in the with c:d ratio <0.3 is 48 as presented in table 1 and numbers table 2 before correction was 10 in young NAION patients and 20 in older NAION patients (total 30). The true number of young NAION patients with c:d <0.3 is 22 of total 32 patients (68.75%) and in older NAION patients it is 26 of total 46 patients (56.5%) . So the total number of patients with c:d <0.3 is (22+26=48) and is 61.5% (48/78) of the total subjects.

Reviewer 2

1. Table 1: The heading on the right column should be" Patients (n=78)". Second row, Age (years, mean± SD). Third row, "Below 50 years (n, %)". Please correct all the others in this table.

Answer: All table headings were revised and corrected as suggested.

2. "+_"should be"±". "<=" should be "≤".

Answer: This was changed as suggested throughout the text.

3. Results part 2, revise the subheading: Visual Outcome in Young NAION Versus Older NAION

Answer: This was changed to “**Visual Outcome in Young vs Older NAION Patients”.**
